# Early noncardiovascular organ failure and mortality in the cardiac intensive care unit

**DOI:** 10.1002/clc.23339

**Published:** 2020-01-30

**Authors:** Jacob C. Jentzer, Brandon Wiley, Courtney Bennett, Dennis H. Murphree, Mark T. Keegan, Ognjen Gajic, Kianoush B. Kashani, Gregory W. Barsness

**Affiliations:** ^1^ Department of Cardiovascular Medicine The Mayo Clinic Rochester Minnesota; ^2^ Division of Pulmonary and Critical Care Medicine, Department of Internal Medicine The Mayo Clinic Rochester Minnesota; ^3^ Department of Health Sciences Research The Mayo Clinic Rochester Minnesota; ^4^ Department of Anesthesiology and Perioperative Medicine The Mayo Clinic Rochester Minnesota; ^5^ Division of Nephrology and Hypertension, Department of Internal Medicine The Mayo Clinic Rochester Minnesota

**Keywords:** cardiac critical care, cardiac intensive care unit, mortality, organ failure, sequential organ failure assessment (SOFA) score

## Abstract

**Background:**

Noncardiac organ failure has been associated with worse outcomes among a cardiac intensive care unit (CICU) population.

**Hypothesis:**

We hypothesized that early organ failure based on the sequential organ failure assessment (SOFA) score would be associated with mortality in CICU patients.

**Methods:**

Adult CICU patients from 2007 to 2015 were reviewed. Organ failure was defined as any SOFA organ subscore ≥3 on the first CICU day. Organ failure was evaluated as a predictor of hospital mortality and postdischarge survival after adjustment for illness severity and comorbidities.

**Results:**

We included 10 004 patients with a mean age of 67 ± 15 years (37% female). Admission diagnoses included acute coronary syndrome in 43%, heart failure in 46%, cardiac arrest in 12%, and cardiogenic shock in 11%. Organ failure was present in 31%, including multiorgan failure in 12%. Hospital mortality was higher in patients with organ failure (22% vs 3%, adjusted OR 3.0, 95% CI 2.5‐3.7, *P* < .001). After adjustment, each failing organ system predicted twofold higher odds of hospital mortality (adjusted OR 1.9, 95% CI 1.1‐2.1, *P* < .001). Mortality risk was highest with cardiovascular, coagulation and liver failure. Among hospital survivors, organ failure was associated with higher adjusted postdischarge mortality risk (*P* < .001); multiorgan failure did not confer added long‐term mortality risk.

**Conclusions:**

Early noncardiovascular organ failure, especially multiorgan failure, is associated with increased hospital mortality in CICU patients, and this risk continues after hospital discharge, emphasizing the need to promote early recognition of organ failure in CICU patients.

**Abbreviations**
APACHE: acute physiology and chronic health evaluation
BMI: body mass index
CCI: Charlson comorbidity index
CI: confidence interval
CICU: cardiac intensive care unit
IABP: intra‐aortic balloon pump
LOS: length of stay
OASIS: Oxford Acute Severity of Illness Score
OR: odds ratio
PAC: pulmonary artery catheter
PCI: percutaneous coronary intervention
SOFA: sequential organ failure assessment

## 1 INTRODUCTION

The contemporary cardiac intensive care unit (CICU) serves an increasingly complex patient population with acute and chronic multiorgan dysfunction and superimposed cardiac pathology.^1^, ^2^, ^3^, ^4^, ^5^ Acute cardiovascular diseases remain an important focus of CICU care, yet noncardiovascular disease processes including extracardiac organ failure are becoming more prevalent in CICU patients.^1^, ^4^, ^5^ Organ failure, particularly multiorgan failure, is a final common pathway of critical illness and an important driver of mortality among CICU patients. In particular, acute kidney injury, and acute respiratory failure are increasingly prevalent and have been associated with higher mortality in CICU populations, emphasizing the importance of early recognition and management of these concomitant acute noncardiovascular illnesses.^1^, ^4^

Organ failure can be quantified using the sequential organ failure assessment (SOFA) score, an illness severity score initially described in patients with sepsis.^6^, ^7^, ^8^ The SOFA score is calculated based on a four‐point functional assessment for each of six organ systems (central nervous, cardiovascular, respiratory, renal, liver, and coagulation); individual SOFA organ subscores ≥3 reflect failure of that organ system.^6^, ^7^, ^8^, ^9^, ^10^, ^11^, ^12^, ^13^ The number and pattern of failing organ systems are associated with mortality in critically ill patients (especially among patients with sepsis).^10^, ^14^ In recent studies, the SOFA score has been examined for prediction of short‐term mortality in CICU populations.^9^, ^13^, ^15^, ^16^ The SOFA score on the first CICU day has very good discrimination for hospital mortality in our CICU population, and even minimal organ dysfunction (total SOFA score ≥ 2 on the first CICU day) was associated with higher in‐hospital and postdischarge mortality.^13^ If simple SOFA‐based definitions of organ failure provide useful mortality risk stratification among CICU patients, this could justify their more frequent use.

To the best of our knowledge, no prior studies have explored the epidemiology or prognostic value of early organ failure in CICU patients. The purpose of this study was to evaluate the short‐ and long‐term mortality associated with organ failure on the first CICU day among unselected CICU patients.

## 2 METHODS

This study (IRB # 16‐000722) was approved by the Mayo Clinic Institutional Review Board under an exception from informed consent as posing minimal risk to patients. This was a historical cohort analysis of patients admitted to the CICU at the Mayo Clinic Hospital, St. Mary's Campus, a tertiary‐care hospital in Rochester, Minnesota.^13^, ^17^ This CICU is a closed 16‐bed ICU which cares for critically ill patients with primary cardiac diseases or significant cardiac comorbidities, but does not admit postoperative cardiac surgery patients. Unique adult patients ≥18 years old admitted to the CICU between January 1, 2007, and December 31, 2015, were included using data from the first CICU admission for each patient.^18^ Patients admitted prior to January 1, 2007, patients still hospitalized on December 31, 2015, and patients who did not provide Minnesota Research Authorization under Minnesota state law were excluded.

Demographic, clinical, and laboratory data and information relating to therapeutic interventions and procedures were collected. SOFA scores and individual organ subscores were electronically generated using the worst physiologic values from the first 24 hours of CICU admission.^11^, ^12^, ^13^, ^16^ Consistent with the established SOFA‐based definition, organ failure was defined as a SOFA organ subscore ≥ 3 for that organ system (Supplemental Table S1).^10^ The acute physiology and chronic health evaluation (APACHE) III score, APACHE IV predicted hospital mortality, and Oxford Acute Severity of Illness Score (OASIS) were generated using data from the first 24 hours of CICU admission.^17^, ^19^, ^20^, ^21^, ^22^ Missing data for calculating the SOFA, APACHE III/IV, and OASIS scores were imputed as normal (score of 0), as is customary for prognostic scoring systems.^11^, ^12^, ^20^ The Charlson comorbidity index (CCI) and individual comorbidities were determined using a previously validated electronic algorithm.^23^ Admission diagnoses were defined as all International Classification of Diseases (ICD)‐9 diagnosis codes recorded on the day of CICU admission and the day before or after CICU admission; these admission diagnoses were not mutually exclusive, and the primary admission diagnosis could not be determined.

The primary endpoint was all‐cause hospital mortality; the secondary endpoints were all‐cause CICU mortality and postdischarge mortality during long‐term follow‐up. Mortality data were extracted electronically from Mayo Clinic databases, the state of Minnesota electronic death certificates, and the Rochester Epidemiology Project database on February 1, 2018, using previously described methodology.^24^ Categorical variables are reported as number (%), and the chi‐squared test was used to compare groups. Continuous variables are reported as mean ± SD; Student's *t* test was used to compare two groups, and analysis of variance was used to compare more than two groups. Logistic regression was performed to assess organ failure as a predictor of hospital mortality before and after adjusting for age, gender, race, APACHE IV predicted hospital mortality, CCI, and admission diagnoses of ACS and heart failure (HF). Kaplan‐Meier analysis was performed to determine postdischarge survival in patients discharged alive from the hospital; groups were compared using the log‐rank test. Cox proportional‐hazards models were used to assess the association of organ failure with postdischarge survival, adjusting for age, gender, race, APACHE IV predicted hospital mortality, CCI, and admission diagnoses of ACS and HF. *P* values <.05 were considered statistically significant. Statistical analyses were performed using JMP version 13.0 Pro (SAS Institute, Cary, North Carolina).

## 3 RESULTS

### 3.1 Study population

We screened 12 904 adult admissions to the CICU during the study period; 2900 patients were excluded (1877 readmissions, 755 patients without Minnesota Research Authorization and 268 patients admitted before or still hospitalized after the study period), yielding an overall study population of 10 004 patients.^13^ Day 1 SOFA score data were available for 9989 (99.9%) patients. Day 1 SOFA organ subscore data were available for >90% of patients for the cardiovascular, coagulation, central nervous system, and renal subscores, but fewer than one‐third of patients had available data to calculate the liver or respiratory subscores (Supplemental Table S1). Patients who did not have data available to calculate an individual SOFA subscore were considered not to have failure of that organ system. The mean age was 67 ± 15 years, and 3746 (37%) patients were female. Admission diagnoses included acute coronary syndrome (ACS) in 4267 (43.1%) patients, HF in 4564 (46.1%) patients, cardiogenic shock in 1078 (10.9%) patients, cardiac arrest in 1193 (12.0%) patients, and sepsis in 605 (6.1%) patients.

### 3.2 Prevalence of organ failure

Early organ failure (any SOFA organ subscore ≥ 3 on the first CICU day) was present in 3102 (31.0%) patients, with a mean of 1.6 failing organ systems. The most common failing organ systems were respiratory and cardiovascular, followed by renal (Supplemental Table S1); coagulation and liver failure were uncommon (fewer than 1% of patients). The majority of patients with organ failure had single‐organ failure (60.3%) or two‐organ failure (25.3%), while only 14.3% had failure of >2 organs. The majority of patients with central nervous system (91.3%) or cardiovascular (74.9%) failure had multiorgan failure. Noncardiovascular organ failure was present in 2820 (28.2%) patients, including 2029 (20.3%) with noncardiovascular single organ failure and 791 (7.9%) with noncardiovascular multiorgan failure.

Patients with single‐organ and multiorgan failure were older and expectedly had greater overall illness severity, higher rates of comorbidities, different rates of therapeutic interventions, and longer LOS than patients without organ failure (Table 1). The majority of patients with multiorgan failure received catecholamines and mechanical ventilation on the first CICU day. Patients with organ failure (especially multiorgan failure) more frequently had admission diagnoses of cardiogenic shock, cardiac arrest, and sepsis; the prevalence of organ failure was likewise higher in patients with these admission diagnoses (Supplemental Figure S1).

**Table 1:** Baseline characteristics and provided therapies for patients without early organ failure and with one or more than one failing organ system on the first CICU day. Data represented as number (% of total) for categorical variables and mean ± SD for continuous variables. *P* value is for chi‐squared test (categorical variables) or analysis of variance (continuous variables) between the three groups

	No organ failure *N* = 6902 (69.0%)	Single organ failure *N* = 1872 (18.7%)	Multiorgan failure *N* = 1230 (12.3%)	*P* value
*Baseline characteristics*				
Age	66.8 ± 15.3	68.9 ± 15.3	68.9 ± 14.2	<.0001
Female	2514 (36.5%)	765 (40.9%)	459 (37.3%)	.0025
BMI	29.3 ± 6.8	29.8 ± 7.4	30.1 ± 7.9	.0002
*Comorbidities*				
CCI	2.1 ± 2.5	3.0 ± 2.8	2.8 ± 2.8	<.0001
Myocardial infarction	1345 (19.6%)	406 (21.8%)	224 (18.2%)	.0383
Chronic heart failure	151 (16.7%)	518 (27.8%)	284 (23.1%)	<.0001
Stroke	803 (11.7%)	269 (14.4%)	157 (12.8%)	.0059
Chronic kidney disease	1133 (16.5%)	563 (30.2%)	331 (27.0%)	<.0001
Diabetes mellitus	1792 (26.1%)	621 (33.3%)	417 (34.0%)	<.0001
Cancer	1404 (20.4%)	444 (23.8%)	282 (23.0%)	.0027
Lung disease	1221 (17.8%)	455 (24.4%)	267 (21.7%)	<.0001
Prior dialysis	165 (2.4%)	243 (13.0%)	162 (13.2%)	<.0001
*Admission diagnoses*				
Acute coronary syndrome	3036 (44.4%)	692 (37.4%)	539 (44.3%)	<.0001
Heart failure	2678 (39.2%)	1106 (59.7%)	780 (64.1%)	<.0001
Cardiac arrest	412 (6.0%)	247 (13.3%)	534 (43.9%)	<.0001
Cardiogenic shock	256 (3.8%)	265 (14.3%)	557 (45.8%)	<.0001
Sepsis	181 (2.6%)	175 (9.4%)	249 (20.5%)	<.0001
*Therapeutics in CICU*				
Inpatient PCI	2587 (37.6%)	488 (26.1%)	348 (28.3%)	<.0001
Invasive ventilation day 1	59 (0.9%)	421 (22.5%)	917 (74.6%)	<.0001
Catecholamines day 1	363 (5.3%)	479 (25.6%)	928 (75.4%)	<.0001
>1 catecholamine day 1	20 (0.3%)	93 (5.0%)	463 (37.6%)	<.0001
IABP in CICU	393 (5.7%)	191 (10.2%)	281 (22.8%)	<.0001
PAC in CICU	372 (5.4%)	140 (7.5%)	209 (17.0%)	<.0001
Blood transfusion in CICU	499 (7.2%)	316 (16.9%)	358 (29.1%)	<.0001
Dialysis in CICU	211 (3.1%)	113 (6.0%)	162 (13.2%)	<.0001
*Severity of illness and LOS*				
APACHE‐III score	51.6 ± 16.9	70.0 ± 19.2	100.0 ± 30.5	<.0001
APACHE‐IV mortality (%)	9.2 ± 9.1	22.0 ± 17.4	52.3 ± 26.7	<.0001
OASIS	21.3 ± 6.9	29.4 ± 9.0	41.7 ± 9.9	<.0001
Day 1 total SOFA score	1.9 ± 1.3	5.4 ± 1.6	10.1 ± 2.7	<.0001
CICU LOS	2.1 ± 4.9	2.9 ± 3.3	3.8 ± 3.9	<.0001
Hospital LOS	6.8 ± 13.1	10.1 ± 12.5	10.8 ± 13.6	<.0001
*Failing organ systems*				
Cardiovascular failure	N/A	282 (15.1%)	842 (68.5%)	<.0001
Central nervous system failure	N/A	54 (2.9%)	566 (46.0%)	<.0001
Coagulation failure	N/A	40 (2.1%)	51 (4.2%)	<.0001
Liver failure	N/A	16 (0.8%)	29 (2.4%)	<.0001
Renal failure	N/A	650 (34.7%)	392 (31.9%)	<.0001
Respiratory failure	N/A	830 (44.3%)	1121 (91.1%)	<.0001

Abbreviations: APACHE, Acute Physiology and Chronic Health Evaluation; BMI, body mass index; CICU, cardiac intensive care unit; IABP, intra‐aortic balloon pump; LOS, length of stay; OASIS, Oxford Acute Severity of Illness Score; PAC, pulmonary artery catheter; PCI, percutaneous coronary intervention; SOFA, Sequential Organ Failure Assessment.

### 3.3 Mortality and organ failure

**Figure 1:**
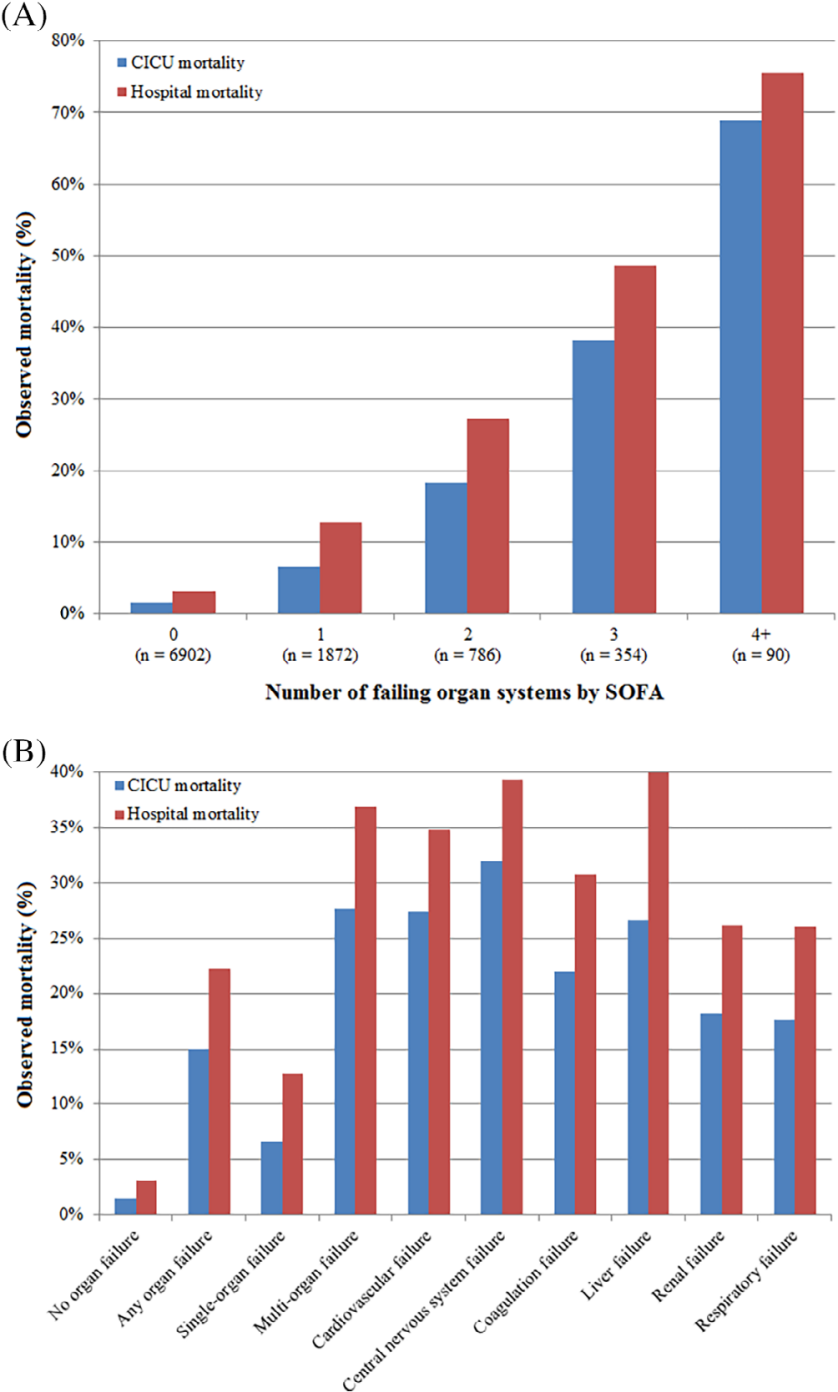
A, Cardiac intensive care unit (CICU) and hospital mortality as a function of the number of early failing organ systems. B, CICU and hospital mortality in patients with failure of each organ system

All‐cause hospital mortality occurred in 908 (9.1%) patients, including CICU mortality in 570 (5.7%) patients. Patients with organ failure had higher hospital mortality (22.3% vs 3.1%, *P* < .001), accounting for 76% of hospital deaths (Figure 1A). Patients with multiorgan failure had even higher hospital mortality (36.9% vs 5.2%, *P* < .001), accounting for 50% of all hospital deaths. There was a stepwise increase in CICU and hospital mortality as a function of the increasing number of failing organ systems (Figure 1B). Hospital mortality occurred in 23.0% of patients with any noncardiovascular organ failure and 40.8% of patients with noncardiovascular multiorgan failure, with a similar stepwise increase in CICU and hospital mortality as a function of increasing number of failing noncardiovascular organ systems.

**Figure 2:**
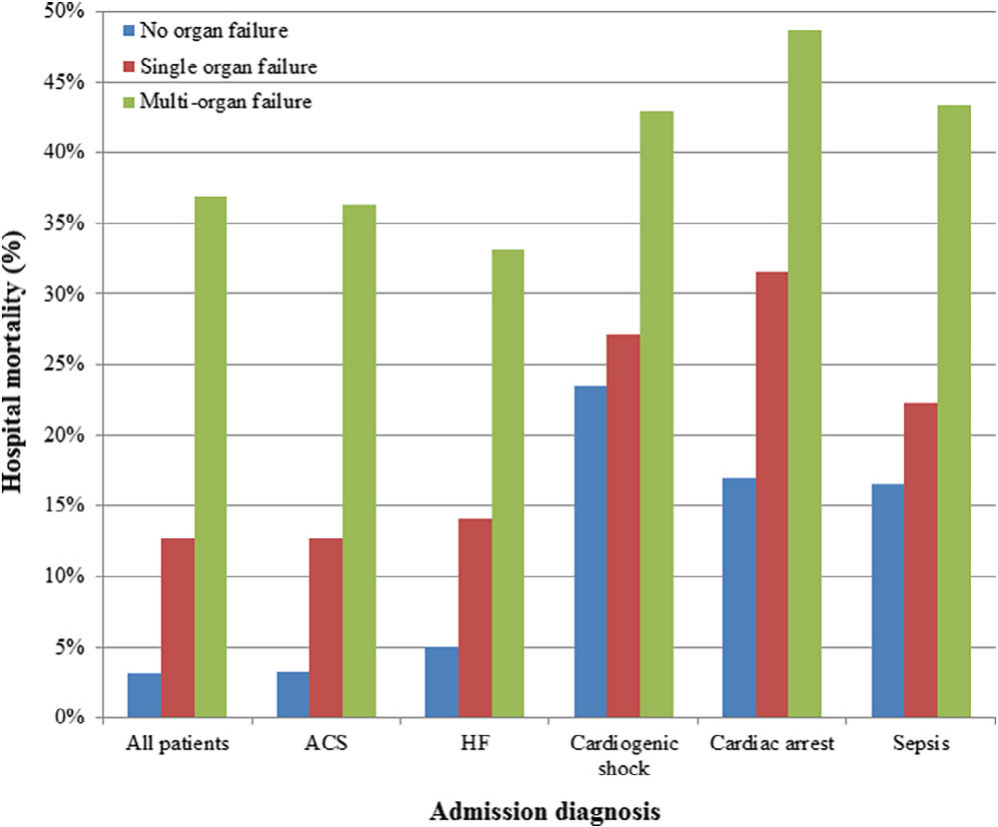
Hospital mortality in patients with single‐organ and multiorgan failure as a function of admission diagnosis

After multivariate adjustment (Table 2), the presence of organ failure was associated with more than threefold higher hospital mortality (adjusted OR 3.02, 95% CI 2.47‐3.68, *P* < .001) and each failing organ system was associated with a twofold higher risk of hospital mortality **(**adjusted OR 1.93, 95% CI 1.74‐2.14, *P* < .001). Hospital mortality among patients with single and multiorgan failure varied as a function of admission diagnosis (Figure 2). Patients with multiorgan failure had high hospital mortality regardless of admission diagnosis, but mortality was higher among patients with cardiogenic shock, cardiac arrest, and sepsis in the absence of multiorgan failure.

**Table 2:** Logistic regression for prediction of hospital mortality before (unadjusted) and after adjusting for age, gender, race, CCI, APACHE‐III score, and admission diagnoses of ACS and HF. OR and 95% CI values are compared to patients without organ failure. *All *P* < .0001 except adjusted OR for central nervous system failure (*P* = .80)

	Unadjusted OR (95% CI)	Adjusted OR (95% CI)
Any organ failure	8.888 (7.576‐10.427)	3.015 (2.473‐3.677)
Single organ failure	4.509 (3.721‐5.463)	2.675 (2.171‐3.294)
Multiorgan failure vs single‐organ failure	18.110 (15.153‐21.643) 4.017 (3.360‐4.802)	4.590 (3.536‐5.957) 1.716 (1.373‐3.145)
Each failing organ system	3.109 (2.907‐3.324)	1.927 (1.738‐2.137)
Cardiovascular failure	8.629 (7.416‐10.040)	2.704 (2.229‐3.280)
Central nervous system failure	8.522 (7.123‐10.197)	0.965 (0.746‐1.250)*
Coagulation failure	4.562 (2.907‐7.158)	2.907 (1.728‐4.888)
Liver failure	6.793 (3.727‐12.383)	3.983 (2.014‐7.874)
Renal failure	4.624 (3.941‐5.427)	1.968 (1.623‐2.386)
Respiratory failure	6.735 (5.840‐7.768)	1.714 (1.403‐2.094)

	Unadjusted		Adjusted	
	OR (95% CI)	*P* value	OR (95% CI)	*P* value
Any organ failure	8.888 (7.576‐10.427)	<.0001	3.015 (2.473‐3.677)	<.0001
Single organ failure	4.509 (3.721–5.463)	<.0001	2.675 (2.171–3.294)	<.0001
Multiorgan failure vs single‐organ failure	18.110 (15.153‐21.643) 4.017 (3.360‐4.802)	<.0001 <.0001	4.590 (3.536‐5.957) 1.716 (1.373‐3.145)	<.0001 <.0001
Each failing organ system	3.109 (2.907‐3.324)	<.0001	1.927 (1.738‐2.137)	<.0001
Cardiovascular failure	8.629 (7.416‐10.040)	<.0001	2.704 (2.229‐3.280)	<.0001
Central nervous system failure	8.522 (7.123–10.197)	<.0001	0.965 (0.746–1.250)	.7896
Coagulation failure	4.562 (2.907–7.158)	<.0001	2.907 (1.728–4.888)	<.0001
Liver failure	6.793 (3.727–12.383)	<.0001	3.983 (2.014–7.874)	<.0001
Renal failure	4.624 (3.941–5.427)	<.0001	1.968 (1.623–2.386)	<.0001
Respiratory failure	6.735 (5.840–7.768)	<.0001	1.714 (1.403–2.094)	<.0001

Abbreviations: ACS, acute coronary syndrome; APACHE, Acute Physiology and Chronic Health Evaluation; CCI, Charlson comorbidity index; CI, confidence interval; HF, heart failure; OR, odds ratio.

CICU and hospital mortality risk in patients with organ failure varied with the organ system involved (Figure 1), being highest for liver, central nervous system, and cardiovascular failure. Unadjusted OR values for hospital mortality were highest for central nervous system and cardiovascular failure (Table 2), while adjusted OR values were highest for liver and coagulation failure, followed by cardiovascular failure (Table 2). Hospital mortality varied among patients with each single‐organ failure and various combinations of two‐organ failure (Supplemental Figures S2 and S3); *P* < .001 between groups.

### 3.4 Postdischarge outcomes

**Figure 3:**
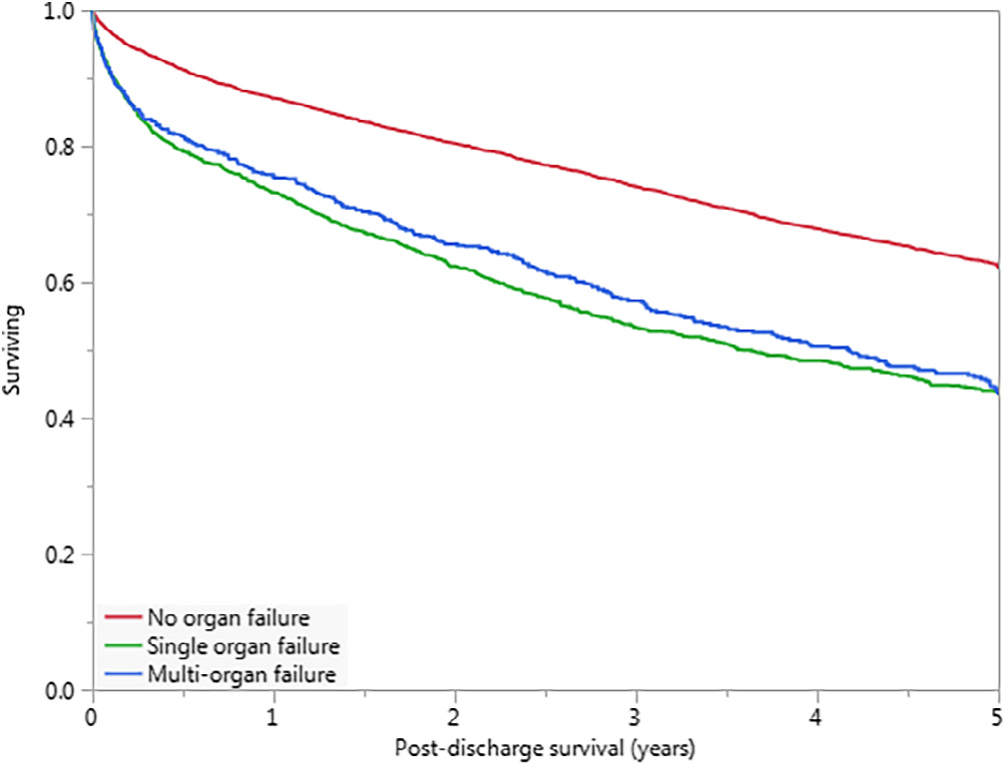
Kaplan‐Meier survival curves for hospital survivors with 1 (green) or ≥2 (blue) organ failures compared to patients without organ failure (red). *P* < .001 by log‐rank for no organ failure vs either single or multiorgan failure; *P* > .1 by log‐rank for single vs multiorgan failure

Postdischarge mortality occurred in 3623 (39.8%) of the 9096 hospital survivors during a mean follow‐up of 3.4 ± 2.9 years; 1284 (14.1%) hospital survivors had follow‐up less than 1 year. Postdischarge survival was lower among the hospital survivors with single or multiorgan failure compared to those without organ failure (Figure 3; *P* < .001 by log‐rank), but was similar between patients with single‐ or multiorgan failure (*P* > .1 by log‐rank). Using Cox proportional‐hazards models, hospital survivors with organ failure (adjusted RR 1.23, 95% CI 1.13‐1.34, *P* < .001) had an increased risk of postdischarge mortality. Hospital survivors with organ failure were less likely to be discharged to home (59.0% vs 80.5%, *P* < .001) and more likely to be discharged to a skilled nursing facility (32.4% vs 15.4%, *P* < .001) (Supplemental Figure S4).

## 4 DISCUSSION

We report the association of early organ failure with hospital and postdischarge mortality in a contemporary CICU population using SOFA‐based organ failure definitions. Organ failure was present in nearly one‐third of patients, the majority of which was noncardiovascular organ failure. Early organ failure was associated with increased mortality at all time points, with a stepwise increase in hospital mortality as the number of failing organ systems increased. Organ failure remained associated with higher hospital mortality after adjustment for the APACHE IV predicted mortality, implying that early organ failure is associated with adverse outcomes beyond standard measures of illness severity. Patients admitted with cardiac arrest, cardiogenic shock, or sepsis had a higher prevalence of organ failure, and the strength of the association between organ failure and hospital mortality differed depending on the admission diagnosis. The hospital mortality risk varied with specific single organ and multiorgan failures, emphasizing the association between particular patterns of organ failure and outcomes in this CICU population. Among hospital survivors, the presence of early organ failure was associated with decreased postdischarge survival, although unexpectedly the presence of multiorgan failure was not worse than single‐organ failure. These results emphasize the importance of organ failure, especially noncardiovascular organ failure, as a contributor to adverse outcomes among CICU patients and validate the utility of simple SOFA‐based organ failure definitions for risk stratification in this population.

In a recent CICU study, acute respiratory failure and acute kidney injury (based on ICD‐9 discharge diagnosis codes) were both independent predictors of hospital mortality after adjusting for illness severity using the OASIS.^4^ Both respiratory failure and renal failure were associated with hospital mortality after adjusting for illness severity in this study, but the risk was lower than with cardiovascular failure. The total SOFA score and individual SOFA subscores on the first CICU day can predict hospital mortality in CICU patients as in general ICU populations.^9^, ^13^, ^15^, ^16^ Patients with SOFA scores <2 on the first CICU day (who are free from organ failure) have a very low risk of mortality during short‐ and long‐term follow‐up.^13^ The present study expands on our prior work by elucidating the association between the number and pattern of organ failures with hospital and postdischarge mortality.^13^, ^16^ Unexpectedly, postdischarge mortality did not differ for patients with multiorgan failure compared to single‐organ failure, potentially reflecting survivor bias given the high hospital mortality rate in this group.

We used the same SOFA‐based organ failure definition as in the seminal study by Moreno et al that established the relationship between organ failure and mortality in critically ill patients.^10^ As in our unadjusted analyses, cardiovascular and central nervous system failure were of primary importance for predicting mortality in the study by Moreno et al.^10^ Notably, the SOFA score was initially developed based on variables that are relevant for patients with sepsis, as opposed to CICU patients.^6^, ^7^, ^8^, ^9^ We observed a stepwise increase in hospital mortality as a function of increasing number of organ failures, similar to a recent study of nearly 1 million patients with sepsis by Shankar‐Hari et al.^14^ As in our study, the mortality risk varied with individual organ failures and specific combinations of organ failure.^14^ A stepwise increase in hospital mortality associated with an increasing number of acute organ failures was observed among patients with cardiogenic shock due to acute myocardial infarction in the Nationwide Inpatient Sample using ICD‐9 discharge diagnoses to define organ failure.^25^ We replicated these results in our broader CICU population using a more objective SOFA‐based definition of organ failure near the time of CICU admission.

In prior studies, the cardiovascular, central nervous system and renal SOFA subscores had the greatest discrimination for hospital mortality.^10^, ^13^, ^16^, ^26^, ^27^ Similarly, cardiovascular and central nervous system failure had the strongest association with mortality in our unadjusted analyses, followed by respiratory and liver failure. Studies in general critically ill patients have demonstrated that the central nervous system SOFA subscore (ie, the Glasgow Coma Scale) carries the greatest prognostic value for short‐term mortality.^26^, ^27^ Toma et al demonstrated that the central nervous system and cardiovascular failure SOFA organ subscores were most important for predicting mortality.^27^ In our study, cardiovascular failure remained a strong predictor of mortality even after adjustment, emphasizing the importance of shock as a determinant of outcomes beyond standard measures of illness severity. We observed an important discrepancy in the associations between central nervous system failure and mortality when comparing adjusted and unadjusted analyses: central nervous system failure had the highest unadjusted OR for hospital mortality, yet was not significantly associated with mortality after adjustment for APACHE‐IV. Rather than excluding a meaningful association between central nervous system failure and mortality in this population, we believe that this paradoxical finding reflects the ability of the APACHE score to effectively account for central nervous system failure.^19^, ^22^ The least frequent organ failures (coagulation and liver) had the strongest adjusted association with mortality, differing from our prior study which concluded that the coagulation and liver SOFA subscores contributed the least to mortality risk stratification.^16^

The association between liver and coagulation system failure and mortality has not been explored in prior studies of CICU patients, and our study highlights the importance of noncardiovascular organ failure among CICU patients. The strong association between coagulation and liver failure and mortality in CICU patients is presumably multifactorial. Coagulation and liver failure were uncommon, suggesting that patients with these specific organ failures were outliers who likely differed from other patients in meaningful ways that contributed to worse outcomes. Notably, the APACHE scoring systems do not incorporate platelet count, although they do include bilirubin, and therefore might not adequately account for the mortality risk conferred by the extreme abnormalities in these variables that characterize liver and coagulation failure.^19^, ^22^ Finally, it is probable that the underlying disease processes producing liver and coagulation failure were themselves associated with adverse outcomes. Sepsis was approximately twice as prevalent in patients with liver and coagulation failure, and the liver and coagulation SOFA subscores were most strongly predictive of mortality in a recent study of patients with sepsis.^28^

As with all retrospective single‐center cohort studies, this study has a number of limitations despite its large sample size, including the potential for unmeasured confounding and bias due to local practice styles. Our patient population may be distinct from other centers, as reflected by a lower hospital mortality rate and a lower rate of acute coronary syndromes.^2^, ^3^, ^4^, ^15^ Data for calculating each individual SOFA subscore were not present for all patients, and data for the respiratory and liver subscores were available in fewer than one‐third of patients. Although these missing data could have potentially influenced our results, our clinical experience is that patients with or at risk for significant organ dysfunction would usually have had laboratory testing to allow calculation of these individual SOFA subscores, minimizing this effect. SOFA‐based organ failure definitions cannot distinguish acute from chronic organ failure and do not consider other potential markers of organ dysfunction.^7^ This study did not evaluate the development of organ failure after the first CICU day. To adjust for illness severity and comorbidities, we used the APACHE IV predicted mortality, which shares some variables with the SOFA score and could have influenced our adjusted mortality results particularly as it relates to central nervous system failure.^6^, ^7^, ^8^, ^17^, ^19^, ^22^ We could not determine the primary admission diagnosis or specific indication for CICU admission. Postdischarge mortality was adjudicated using electronic review of health records for notification of patient death, which may be less accurate than methods such as the Social Security Death Index.

## 5 CONCLUSIONS

Early organ failure based on the SOFA score is common in CICU patients and associated with higher hospital and postdischarge mortality. The number and pattern of failing organs are associated with short‐term mortality. Our findings emphasize the importance of organ failure as a predictor of mortality in CICU patients and highlight the persistent long‐term mortality hazard faced by patients who develop organ failure during their early CICU course. Patients with noncardiovascular organ failure, particularly less‐frequent organ failure such as liver and coagulation, fared the worst. These simple and clinically relevant SOFA‐based definitions of organ failure can be easily applied at the bedside by CICU practitioners and may be useful endpoints for future clinical trials in CICU populations.

## CONFLICT OF INTEREST

The authors declare that there are no relevant financial disclosures or conflicts of interest related to this work.

## Supporting information


**Appendix** **S1** Supporting Information
