# Supplementary material for: Early noncardiovascular organ failure and mortality in the cardiac intensive care unit
Source: Clin Cardiol. 2020 Jan 30;43(5):516–23. doi: 10.1002/clc.23339 (PMC7244298; doi:10.1002/clc.23339)

Appendix 1. This appendix was part of the submitted manuscript and has been peer reviewed. It is posted as supplied by the authors.

Early Non-Cardiovascular Organ Failure and Mortality in the Cardiac Intensive Care Unit

Jacob C. Jentzer, MD; Brandon Wiley, MD; Courtney Bennett, DO; Dennis H. Murphree, Ph.D; Mark T. Keegan, MB MRCPI;

Ognjen Gajic, MD; Kianoush B. Kashani, MD MS; Gregory W. Barsness, MD

All from the Mayo Clinic, 200 First Street SW, Rochester MN 55905

Page 2: Supplemental Table 1

Page 3: Supplemental Figure 1

Page 4: Supplemental Figure 2

Page 5: Supplemental Figure 3

Page 6: Supplemental Figure 4

Supplemental Table 1: Definitions for organ failure in each organ system based on individual SOFA organ sub-score value ≥3 for that organ system, in addition to the availability of data to calculate each individual SOFA organ sub-score and the prevalence of failure of each organ system. Data presented as n (%). Worst physiologic values for each of the 6 organ sub-scores are used for a given day to calculate the individual SOFA organ sub-score for that day. Simultaneous PaO_2 and FiO_2 values are used to calculate the PaO_2:FiO_2 (PF) ratio for the respiratory SOFA sub-score. The Glasgow Coma Scale (GCS) used to calculate the central nervous system SOFA sub-score is only obtained when patients are not on sedative medications. Adapted from Vincent JL, et al. *Intensive Care Med*. 1996;22:707-10 and Moreno R, et al. *Intensive Care Med* 1999;25:686-96.

Organ system	Organ failure definition	Availability of data to calculate	Prevalence of organ failure
**Cardiovascular:**<br>Vasopressors (for ≥1 hour)	Dopamine >5 mcg/kg/min, or any epinephrine or norepinephrine	9971 (99.7%)	1142 (11.4%)
**Central nervous system:**<br>Glasgow Coma Scale (GCS)	≤9	9674 (96.7%)	620 (6.2%)
**Coagulation:**<br>Platelet count x 10^3/mm^3	≤50	9300 (93.0%)	91 (0.9%)
**Liver:**<br>Serum bilirubin (mg/dl)	≥6.0	2651 (26.5%)	45 (0.5%)
**Renal:**<br>Serum creatinine (mg/dl) and urine output (UOP, ml/day)	≥3.5 or UOP <500	9431 (94.3%)	1042 (10.4%)
**Respiratory:**<br>Arterial PaO_2:FiO_2 (PF) ratio	≤200	3128 (31.3%)	1951 (19.5%)

Supplemental Figure 1: Prevalence of single and multi-organ failure as a function of admission diagnosis. Admission diagnoses are not mutually-exclusive. ACS, acute coronary syndrome; HF, heart failure.

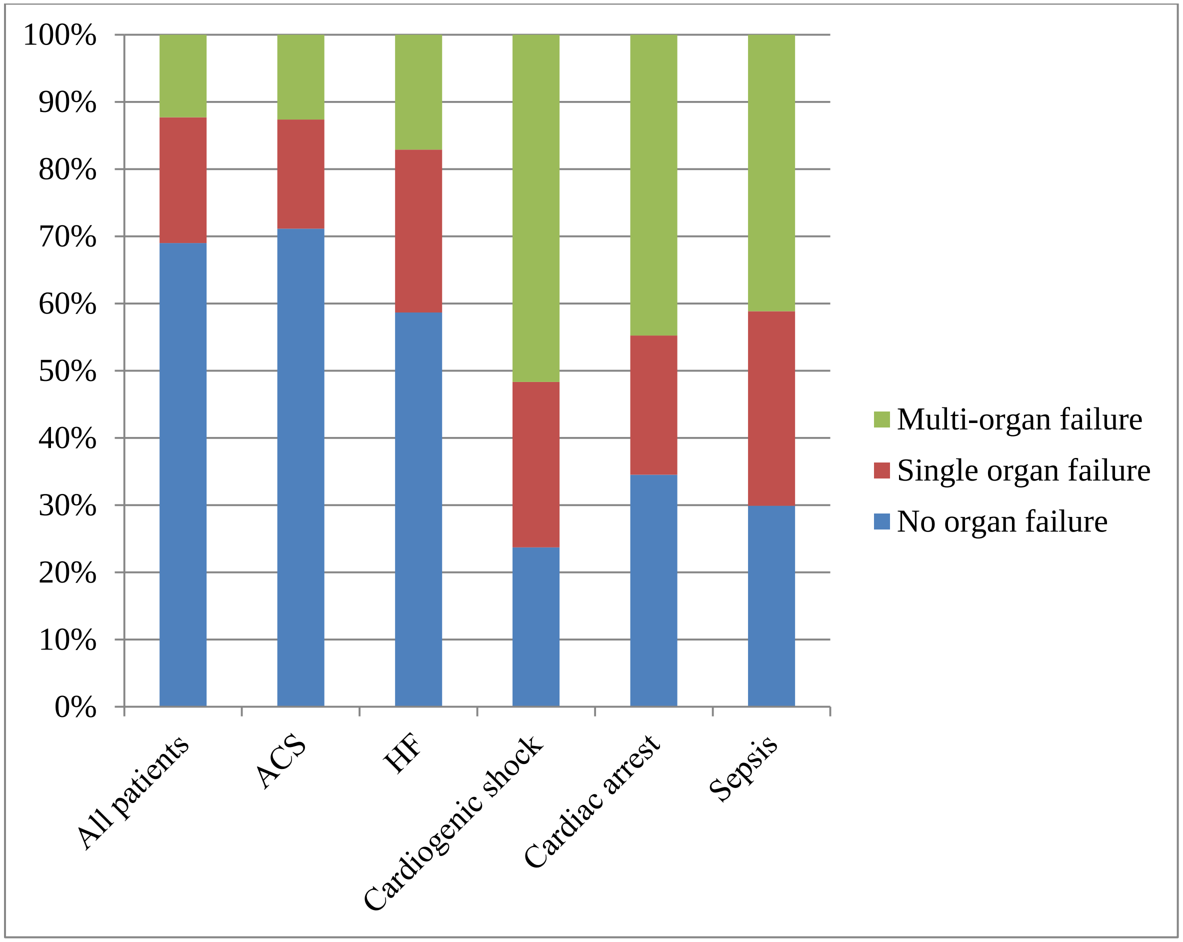

Supplemental Figure 2: Hospital mortality in patients with single-organ failure of each organ system and various combinations of multi-organ failure. Multi-organ failure groups with n < 5 are combined in the "Other 2-organ failure" category. P <0.001 for comparison of mortality between groups.

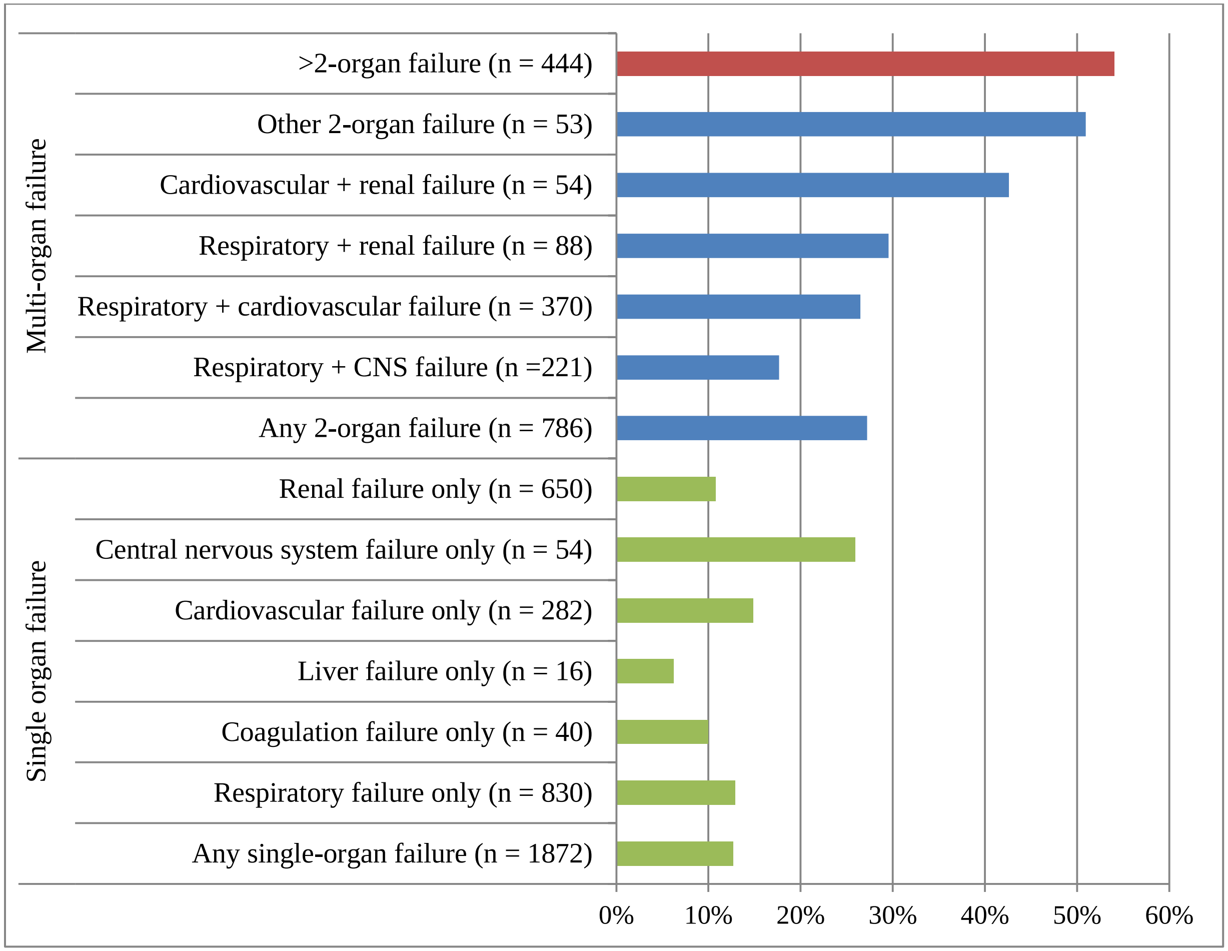

Supplemental Figure 3: Hospital mortality in patients with failure of each organ system, separated by single-organ failure vs. multi-organ failure (i.e. failure of that organ system plus ≥1 other organ system; these groups are not mutually-exclusive).

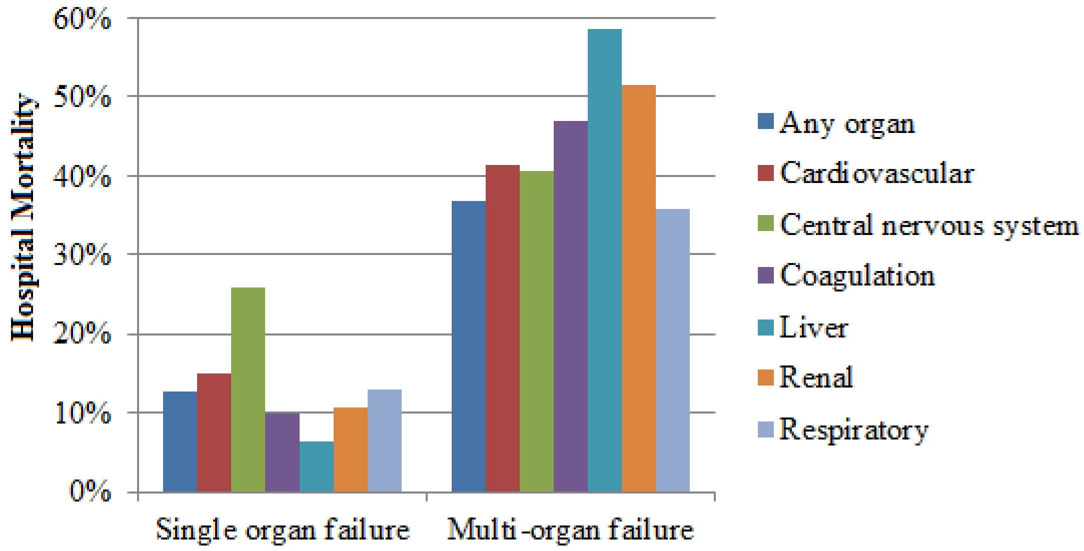

Supplemental Figure 4: Discharge disposition among hospital survivors, separated by the presence of single-organ failure vs. multi-organ failure.

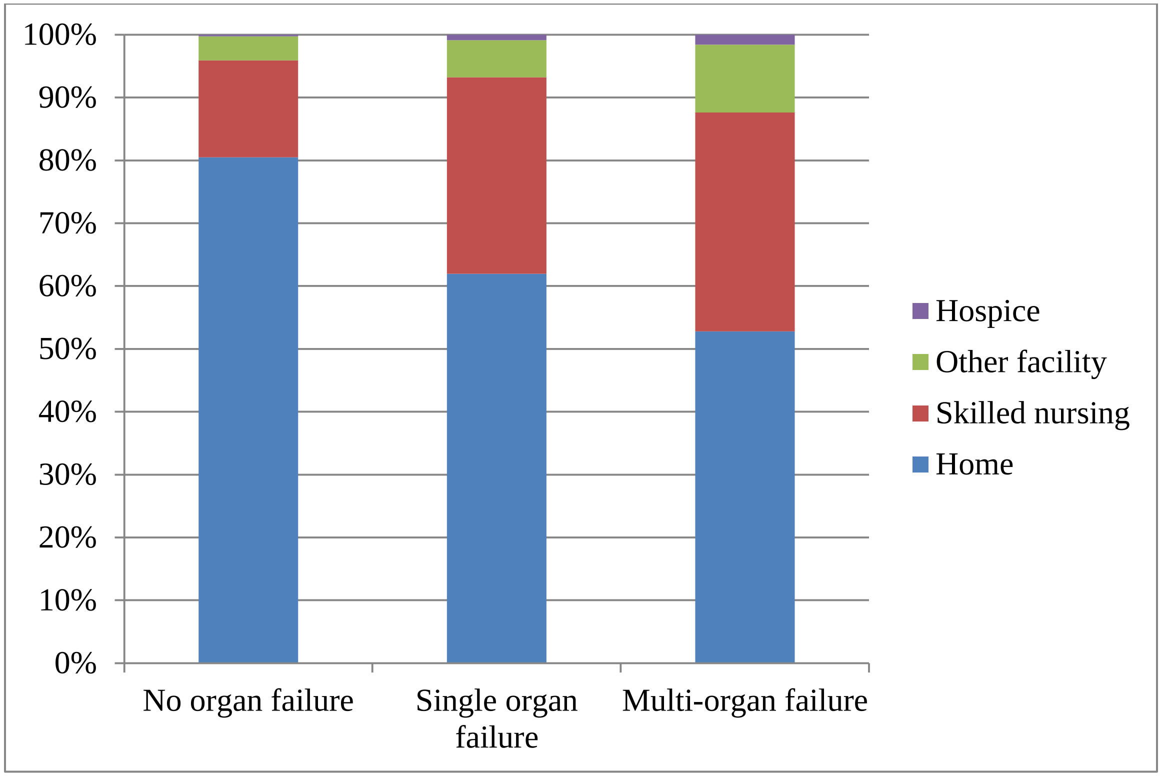

Supplement: Supplementary file 1 — Appendix S1 Supporting Information [file CLC-43-516-s001.pdf]
